# Association Between Climatic Factors and Varicella Incidence in Wuxi, East China, 2010-2019: Surveillance Study

**DOI:** 10.2196/62863

**Published:** 2024-10-02

**Authors:** Kehong Zhang, Ganglei Shen, Yue Yuan, Chao Shi

**Affiliations:** 1 Department of Public Health Wuxi Hospital Affiliated to Nanjing University of Chinese Medicine Wuxi China; 2 President Office Wuxi Hospital Affiliated to Nanjing University of Chinese Medicine Wuxi China; 3 Department of Acute Infectious Disease Wuxi Center for Disease Control and Prevention Wuxi China

**Keywords:** varicella, meteorological factors, Generalized Additive Model, Segmented Linear Regression Model, China, meteorology, regression, statistics, surveillance

## Abstract

**Background:**

Varicella is a common infectious disease and a growing public health concern in China, with increasing outbreaks in Wuxi. Analyzing the correlation between climate factors and varicella incidence in Wuxi is crucial for guiding public health prevention efforts.

**Objective:**

This study examines the impact of meteorological variables on varicella incidence in Wuxi, eastern China, from 2010 to 2019, offering insights for public health interventions.

**Methods:**

We collected daily meteorological data and varicella case records from January 1, 2010, to December 31, 2019, in Wuxi, China. Generalized cross-validation identified optimal lag days by selecting those with the lowest score. The relationship between meteorological factors and varicella incidence was analyzed using Poisson generalized additive models and segmented linear regression. Subgroup analyses were conducted by gender and age.

**Results:**

The study encompassed 64,086 varicella cases. Varicella incidence in Wuxi city displayed a bimodal annual pattern, with peak occurrences from November to January of the following year and lower peaks from May to June. Several meteorological factors influencing varicella risk were identified. A decrease of 1°C when temperatures were ≤20°C corresponded to a 1.99% increase in varicella risk (95% CI 1.57-2.42, *P*<.001). Additionally, a decrease of 1°C below 22.38°C in ground temperature was associated with a 1.36% increase in varicella risk (95% CI 0.96-1.75, *P*<.001). Each 1 mm increase in precipitation above 4.88 mm was associated with a 1.62% increase in varicella incidence (95% CI 0.93-2.30, *P*<.001). A 1% rise in relative humidity above 57.18% increased varicella risk by 2.05% (95% CI 1.26-2.84, *P*<.001). An increase in air pressure of 1 hPa below 1011.277 hPa was associated with a 1.75% rise in varicella risk (95% CI 0.75-2.77, *P*<.001). As wind speed and evaporation increased, varicella risk decreased linearly with a 16-day lag. Varicella risk was higher with sunshine durations exceeding 1.825 hours, with a 14-day lag, increasing by 1.30% for each additional hour of sunshine (95% CI 0.62-2.00, *P*=.006). Subgroup analyses revealed that teenagers and children under 17 years of age faced higher varicella risks associated with temperature, average ground temperature, precipitation, relative humidity, and air pressure. Adults aged 18-64 years experienced increased risk with longer sunshine durations. Additionally, males showed higher varicella risks related to ground temperature and air pressure compared with females. However, no significant gender differences were observed regarding varicella risks associated with temperature (male: *P*<.001; female *P*<.001), precipitation (male: *P*=.001; female: *P*=.06), and sunshine duration (male: *P*=.53; female: *P*=.04).

**Conclusions:**

Our preliminary findings highlight the interplay between varicella outbreaks in Wuxi city and meteorological factors. These insights provide valuable support for developing policies aimed at reducing varicella risks through informed public health measures.

## Introduction

Varicella is a common infectious disease and a significant public health concern in China and globally. According to World Health Organization (WHO) data, the global incidence of varicella and its annual economic burden are increasing year by year [1], and China is facing similar challenges [2,3]. Varicella is an acute infectious disease caused by the varicella-zoster virus (VZV) [4], known for its high infectivity. The prevalence rate of the disease among close contacts is approximately 90% [5]. Transmission primarily occurs through skin contact and the respiratory tract. Symptoms typically include fever, papules on the skin and mucosa, vesicles, and subsequent scabbing, with a characteristic centripetal rash distribution [6,7]. Most cases of varicella are benign and self-limiting, though severe infections can lead to complications such as secondary bacterial infections, disseminated varicella, pneumonia, hepatitis, and, rarely, mortality. In milder cases, recurrence after recovery may manifest as herpes zoster [8]. Following VZV infection, patients typically experience an incubation period of 9-21 days before the onset of symptoms. Susceptible populations include infants, young children, preschoolers, pregnant women, and immunocompromised individuals. Varicella outbreaks commonly occur in settings such as primary schools, kindergartens, and secondary schools, highlighting the increased incidence in collective environments [9].

Several studies have explored the correlation between weather fluctuations and the prevalence of infectious diseases [10]. The impact of climatic factors on the occurrence and spread of illnesses such as influenza [11]; hand, foot, and mouth disease [12]; and tuberculosis [13] has garnered increasing attention as potential early indicators of epidemics. A growing body of evidence highlights the close relationship between climatic variables and the proliferation and incidence of infectious diseases [14]. Previous studies have shown that varicella incidence tends to rise rapidly, often influenced by seasonal fluctuations. Notably, research conducted in Jinan [15] and Wuhan [16], China, identified a bimodal seasonal pattern with more pronounced peaks in winter and smaller peaks in spring. In Takahashi, Japan, an irregular bimodal seasonal pattern was observed, while Okinawa exhibited a unimodal pattern extending from winter to spring [17]. These findings suggest a potential role of climatic factors in varicella transmission, highlighting the need for further investigation into the relationship between these factors and varicella risk to better predict future incidence trends.

In recent years, the incidence of varicella outbreaks in Wuxi has steadily increased, rising from 32.34 per 100,000 individuals in 2010 to 208.05 per 100,000 in 2018, representing a 6-fold increase over 9 years. However, following the introduction of a 2-dose varicella vaccination program for eligible permanent residents in 2018, the incidence of varicella dropped to 174.04 per 100,000 in 2019, indicating a positive impact of vaccination on reducing varicella transmission. Nonetheless, the overall varicella incidence in Wuxi remains significantly higher than the provincial average for Jiangsu Province, where the incidence in 2019 was 147.56 per 100,000. In particular, collective settings such as day care facilities and primary and secondary schools frequently experience cluster outbreaks, posing not only physical health risks to students but also disrupting educational continuity within schools. This study aims to delineate the prevalence of varicella in Wuxi city, located in the humid monsoon climate zone of the northern subtropical region, from 2010 to 2019. Additionally, it seeks to investigate the relationship between varicella incidence and various meteorological factors, thereby providing decision-making support for varicella prevention and control efforts.

## Methods

### Study Area

Wuxi, located in East China in the southern region of Jiangsu Province, lies within the Yangtze River Delta. It borders the Yangtze River to the north and Taihu Lake to the south. Characterized by a northern subtropical humid monsoon climate, Wuxi covers a total area of 4627.46 km^2^. As of the end of 2019, Wuxi city had a permanent resident population of 6.5915 million.

### Varicella Data

The daily varicella case data for Wuxi from January 1, 2010, to December 31, 2019, were sourced from the Wuxi Center for Disease Control and Prevention (CDC). Wuxi city follows a comprehensive infectious disease reporting protocol in compliance with the National Infectious Disease Surveillance System (NDSS) requirements. Medical facilities are required by law to report varicella cases online within 24 hours, providing details such as gender, age, diagnosis date, and hospital visitation information. Stringent data collection, reporting, and inspection procedures ensure the quality of the data. Since June 1, 2017, varicella has been classified as a class C infectious disease under the management of Jiangsu Province. Varicella case data are collected and counted based on the name of the disease, rather than its classification. Therefore, the change in the classification of varicella as an infectious disease before and after June 1, 2017, does not affect the statistical results.

### Meteorological Data

Meteorological data for Wuxi city from January 1, 2010, to December 31, 2019, including temperature (°C), average surface temperature (°C), relative humidity (%), atmospheric pressure (hPa), wind speed (m/s), rainfall (mm), evaporation (mm), and sunshine duration (h), were sourced from the Wuxi City Meteorological Bureau.

### Statistical Analysis

The data were organized using MS Excel 2016 (Microsoft Office), and all data analysis was performed using R 3.6.2 (The R Foundation for Statistical Computing) with a significance level of α=.05. Quantitative variables were described statistically using the mean (SD) and quantiles (0%, 25%, 50%, 75%, and 100%). Count data were described statistically in terms of frequency and composition ratio, that is, n (%). A time series graph illustrating varicella case numbers and fluctuations in meteorological factors was generated. Additionally, Spearman correlation analysis was conducted to assess the relationships between variables.

The Generalized Additive Model (GAM) is a widely used approach for investigating the influence of meteorological factors on health outcomes. For instance, in a multicenter study, the GAM was used to estimate the risk of pulmonary tuberculosis associated with meteorological factors in eastern China. The study revealed that low temperatures, relatively high wind speeds, and low relative humidity contribute to an increased risk of pulmonary tuberculosis [18]. In Hong Kong, GAM analysis revealed that both rotavirus and norovirus hospitalizations were closely related to recent precipitation changes, although in opposite directions [19]. To our knowledge, this is the first study to apply the Poisson GAM and the Piecewise Linear Regression Model to explore the relationship between 7 climate variables and the incidence of varicella in Wuxi, in East China’s Huadong Region.

The GAM incorporates the following covariates: First, a smoothing term is generated using a thin plate spline function with a maximum degree of freedom of 6 to account for long-term trends in the disease number sequence. Second, the number of cases from the previous day to address short-term autocorrelation in the incidence sequence. Third, a binary indicator for holidays, including weekends and statutory holidays. Fourth, seasonal variables to manage the seasonal fluctuations in disease incidence. Subsequently, we compared the generalized cross-validation scores of models across varying lag durations and identified the lag durations with the minimum score as the optimal one for subsequent analysis [20]. Following this, exposure-response curves were plotted to illustrate the relationship between meteorological factors and varicella incidence risk at the optimal lag durations. A segmented regression function was used to assess the magnitude of change in the effect of unit changes in meteorological factors. Additionally, subgroup analysis was conducted to evaluate the robustness of the association. The effect size was quantified by the percentage change in varicella incidence risk, along with its corresponding 95% CI, for every 1-unit increase in meteorological factors.

### Ethical Considerations

This study was approved by the Institutional Review Board of Wuxi Hospital Affiliated with Nanjing University of Chinese Medicine (2024 (paper)-031-01). The study utilizes varicella monitoring data from the Wuxi Center for Disease Control and Prevention, which have been anonymized to ensure effective protection of patient privacy.

## Results

### Data Description

Table 1 presents the summary statistics of meteorological factors and varicella cases in Wuxi city from January 1, 2010, to December 31, 2019. A total of 64,086 cases of varicella were reported during the study period. Males accounted for 54.16% (n=34,709 cases), while females accounted for 45.84% (n=29,377 cases). Teenagers, children, and infants under the age of 17 years comprised the largest group, representing 78.37% (n=50,221 cases) of the total reported cases.

**Figure 1:**
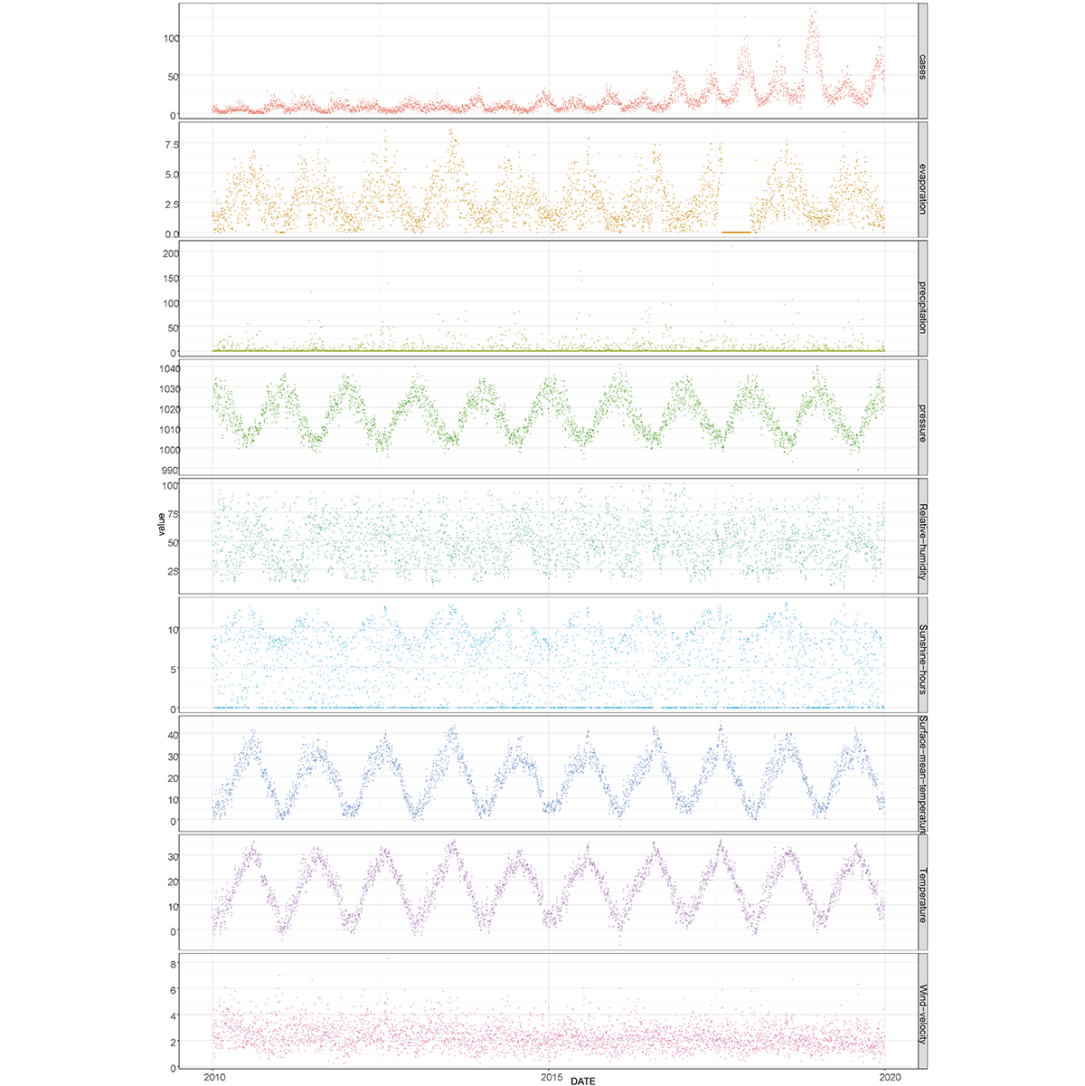
Time distribution of daily incidence of varicella and meteorological factors in Wuxi city between 2010 and 2019.

**Figure 2:**
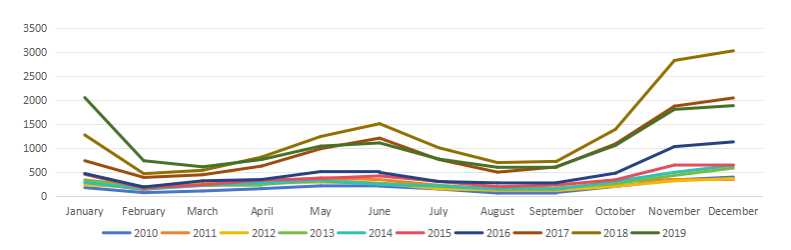
Temporal changes in the incidence rate of varicella.

Figure 1 illustrates the time distribution of daily varicella cases and meteorological variables from 2010 to 2019. According to the monthly chart of varicella incidence between 2010 and 2019 (Figure 2), the highest incidence of varicella occurs from November to January of the following year, accounting for 42.54% (27,261/64,086) of all cases. By contrast, May to June shows a smaller peak, with reported cases making up 18.11% (11,605/64,086) of the total cases.

**Figure 3:**
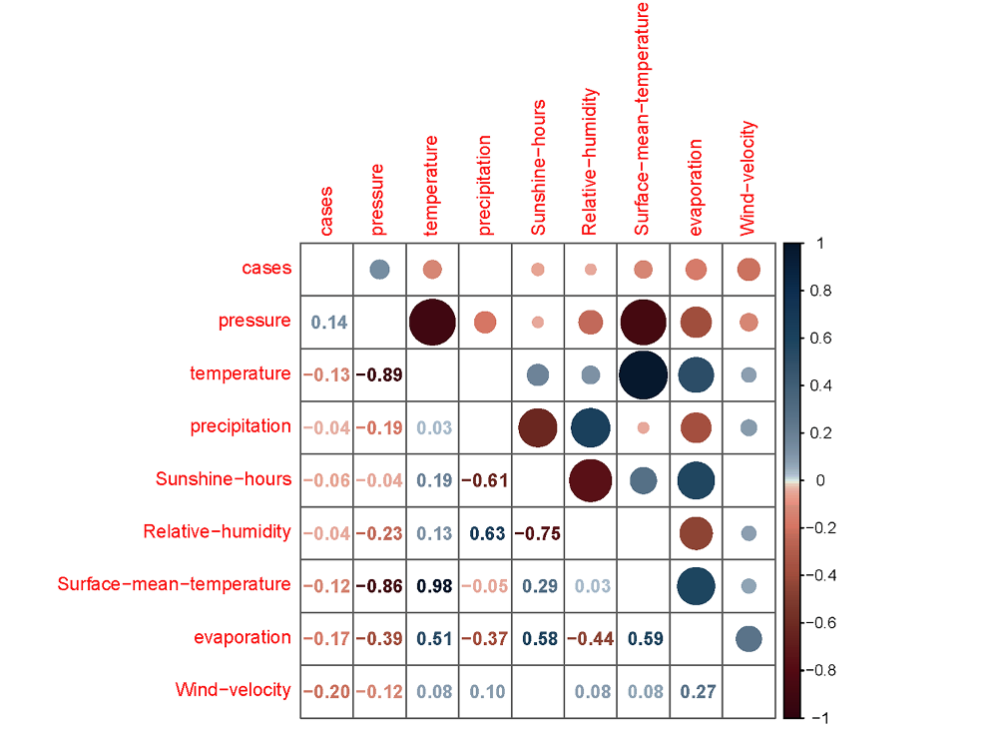
Correlation coefficient between varicella cases and meteorological factors.

According to the Spearman correlation coefficient (Table 2) and the Spearman matrix pie chart (Figure 3) of meteorological factors and varicella cases in Wuxi from 2010 to 2019, the Spearman correlation coefficient between air pressure and varicella cases is 0.145. This indicates a positive correlation, suggesting that the number of varicella cases tends to increase with higher atmospheric pressure. Conversely, temperature, average surface temperature, sunshine duration, evaporation, and wind speed exhibit negative correlations with varicella cases, with coefficients of −0.130, −0.124, −0.058, −0.169, and −0.202, respectively. This suggests that as these meteorological factors increase, the number of varicella cases tends to decrease.

**Figure 4:**
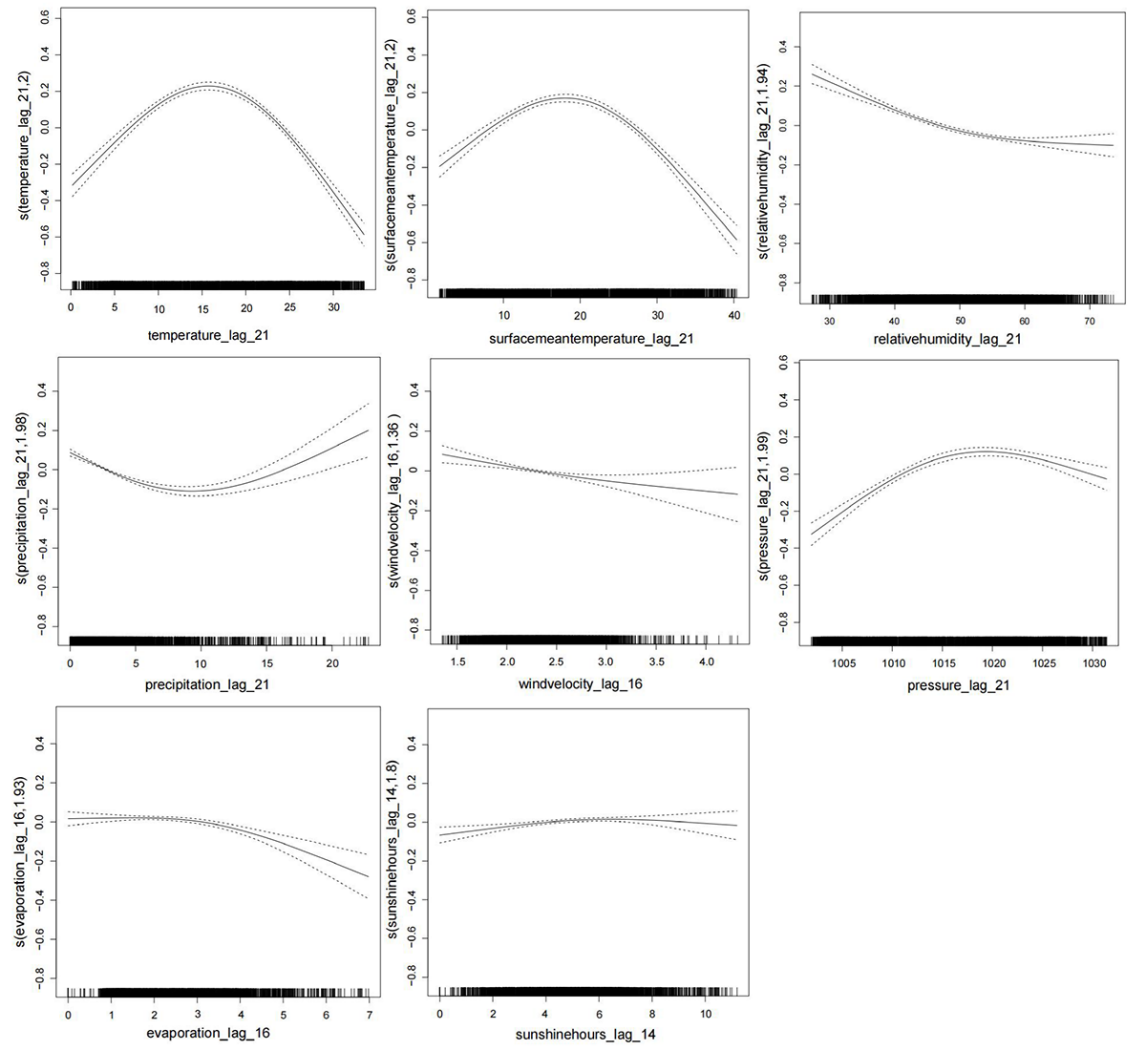
Curve between the smooth varicella curve fitted by GAM and meteorological factors.

We compared the generalized cross-validation scores of models with different lag days and selected the lag days corresponding to the minimum score as the optimal lag days (Table 3). We then plotted the exposure-response curve between meteorological factors and the risk of varicella incidence for the optimal lag days (Figure 4) and used a segmented regression function to evaluate the magnitude of effect changes for unit changes in meteorological factors. Subgroup analysis was conducted to assess the robustness of the association (Table 4).

**Table 1:** Summary statistics of meteorological factors and varicella cases in Wuxi city between 2010 and 2019.

Variable and classification		n (%)	Mean (SD)	Median	P25-P75	Range
Total		64,086	17.55 (18.88)	11	6-21	0 to 136
**Gender**						
	Male	34,709 (54.16)	9.50 (10.30)	6	3-12	0 to 78
	Female	29,377 (45.84)	8.04 (9.07)	5	2-10	0 to 67
**Age**						
	Young (≤ 17 years)	50,221 (78.37)	13.75 (15.63)	9	5-16	0 to 119
	Adults (18-64 years)	13,846 (21.61)	3.79 (4.04)	2	1-5	0 to 27
	Older adults (≥65 years)	19 (0.03)	0.01 (0.07)	0	0-0	0 to 2
**Meteorology measure**						
	Mean temperature (°C)	N/A^a^	17.11 (9.23)	18	9-24.6	–6.1 to 36
	Relative humidity (%)	N/A	49.27 (18.74)	48	35-63	8 to 100
	Wind velocity (m/s)	N/A	2.32 (0.93)	2.2	1.6-2.8	0.2 to 8.3
	Sunshine hours (h)	N/A	4.91 (4.14)	5.2	0-8.6	0 to 13.2
	Precipitation (mm)	N/A	3.53 (11.23)	0	0-1.3	0 to 211.3
	Surface mean temperature (°C)	N/A	19.11 (10.47)	19.9	9.5-27.3	–3.2 to 45.6
	Evaporation (mm)	N/A	2.40 (1.71)	2.1	1.1-3.5	0 to 8.8
	Pressure (hPa)	N/A	1016.20 (9.21)	1016.4	1008.2-1023.3	989.1 to 1041

^a^N/A: not applicable.

**Table 2:** Spearman-level correlation coefficient between meteorological factors and varicella cases in Wuxi city between 2010 and 2019.

Correlation	Pressure	Temperature	Precipitation	Sunshine hours	Relative humidity	Surface mean temperature	Evaporation	Wind velocity	Cases
Pressure	1.000	—^a^	—	—	—	—	—	—	—
Temperature	–0.894	1.000	—	—	—	—	—	—	—
Precipitation	–0.187	0.034	1.000	—	—	—	—	—	—
Sunshine hours	–0.045	0.186	–0.613	1.000	—	—	—	—	—
Relative humidity	–0.230	0.129	0.628	–0.753	1.000	—	—	—	—
Surface mean temperature	–0.864	0.984	–0.045	0.287	0.035	1.000	—	—	—
Evaporation	–0.387	0.514	–0.372	0.575	–0.442	0.586	1.000	—	—
Wind velocity	–0.122	0.082	0.099	–0.005	0.081	0.078	0.265	1.000	—
Cases	0.145	–0.130	–0.037	–0.058	–0.045	–0.124	–0.169	–0.202	1.000

^a^Not applicable.

**Table 3:** Lag days corresponding to the minimum generalized cross-validation score.

Meteorological variables	Lag days	Generalized cross-validation
Pressure	21	1.8255
Temperature	21	1.6707
Precipitation	21	1.8339
Sunshine hours	14	1.8741
Relative humidity	21	1.8019
Surface mean temperature	21	1.7446
Evaporation	16	1.8667
Wind velocity	16	1.8720

**Table 4:** Percentage change in the risk of varicella, its 95% CI, and *P* value.

Variable	Temperature≤20°C	Temperature>20°C
Total	1.99% (1.57 to 2.42), *P*<.001	−3.21% (−3.8 to 2.62), *P*<.001
Male	2.00% (1.48 to 2.53), *P*<.001	−3.19% (−3.96 to 2.41), *P*<.001
Female	1.98% (1.43 to 2.54), *P*<.001	−3.24% (−4.06 to 2.41), *P*<.001
≤17 years	2.50% (2.00 to 3.00), *P*<.001	−4.89% (−5.58 to 4.19), *P*<.001
18-64 years	0.25% (−0.53 to 1.02), *P*=.53	1.61% (0.42 to 2.80), *P*=.008

### Data Analysis

#### Temperature and the Risk of Varicella Incidence

With a 21-day lag, the exposure-response curve illustrating the relationship between temperature and varicella risk shows a nonlinear pattern. As temperature rises, the risk of varicella initially increases and then decreases, with this correlation proving statistically significant (*P*<.001). Consequently, a segmented linear regression function was used to assess the impact of temperature on varicella risk, identifying a cutoff value of 20°C. Subsequent analysis showed that when the temperature was ≤20°C, each 1°C decrease resulted in a 1.99% increase in varicella risk (95% CI 1.57-2.42, *P*<.001). Conversely, when the temperature exceeded 20°C, each 1°C increase resulted in a 3.21% decrease in varicella risk (95% CI −3.80 to 2.62, *P*<.001), which was statistically significant. Gender and age subgroup analyses revealed similar trends in the impact of temperature changes on varicella risk. Specifically, for children under 17 years, when the temperature was ≤20°C, each 1°C decrease led to a 2.50% increase in varicella risk (95% CI 2.00-3.00, *P*<.001; Table 4).

#### Pressure and the Risk of Varicella Incidence

With a 21-day lag, the exposure-response curve illustrating the relationship between air pressure and varicella risk shows a nonlinear pattern. As air pressure increases, the risk of varicella initially rises and then declines, with this correlation proving statistically significant (*P*<.001). Consequently, a segmented linear regression function was used to assess the impact of air pressure on varicella risk, identifying a cutoff value of 1011.28 hPa. Further analysis revealed that when the air pressure was ≤1011.28 hPa, each 1 hPa decrease led to a 1.75% increase in varicella risk (95% CI 0.75-2.77, *P*<.001). However, when the air pressure exceeded 1011.28 hPa, each 1 hPa increase resulted in a 0.08% decrease in varicella risk (95% CI −0.48 to 0.32, *P*=.69), but this association was not significant. Gender and age subgroup analyses indicated that when the air pressure was ≤1011.28 hPa, the risk of varicella increased more in males compared with females. Additionally, the impact on children under 17 years (*P*<.001) was significantly greater than that on adults aged 18-64 years (*P*<.001; Table 5).

**Table 5:** Percentage change in the risk of varicella, its 95% CI, and *P* value.

Variable	Pressure≤1011.28 hPa	Pressure>1011.28 hPa
Total	1.75% (0.75 to 2.77), *P*<.001	−0.08% (−0.48 to 0.32), *P*=.69
Male	2.10% (0.81 to 3.40), *P*=.001	0.18% (−0.31 to 0.67), *P*=.48
Female	1.33% (−0.03 to 2.72), *P*=.06	−0.40% (−0.92 to 0.12), *P*=.13
≤17 years	3.61% (2.39 to 4.84), *P*<.001	−0.25% (−0.72 to 0.22), *P*=.29
18-64 years	−3.40% (−5.19 to 1.59), *P*<.001	0.52% (−0.19 to 1.24), *P*=.15

#### Precipitation and the Risk of Varicella Incidence

With a 21-day lag, the exposure-response curve illustrating the relationship between precipitation and varicella risk shows a nonlinear pattern. As precipitation increases, the risk of varicella initially decreases and then increases, with this correlation being statistically significant (*P*<.001). Consequently, a segmented linear regression function was used to assess the impact of precipitation on varicella incidence risk, identifying a cutoff value of 4.88 mm. Further analysis reveals that when precipitation is ≤4.88 mm, each 1 mm decrease results in a 4.51% decrease in varicella incidence risk (95% CI −5.43 to 3.59, *P*<.001). Conversely, when precipitation exceeds 4.88 mm, each 1 mm increase leads to a 1.62% increase in varicella incidence risk (95% CI 0.93-2.30, *P*<.001). Analysis of gender and age subgroups indicates that precipitation has a greater impact on varicella risk in males compared with females, and its effect is more pronounced in children under 17 years than in adults aged 18-64 years (Table 6).

**Table 6:** Percentage change in the risk of varicella, its 95% CI, and *P* value.

Variable	Precipitation≤4.88 mm	Precipitation>4.88 mm
Total	−4.51% (−5.43 to −3.59), *P*<.001	1.62% (0.93 to 2.30), *P*<.001
Male	−5.09% (−6.20 to −3.96), *P*<.001	1.65% (0.75 to 2.53), *P*<.001
Female	−3.68% (−4.86 to −2.48), *P*<.001	1.60% (0.66 to 2.54), *P*<.001
≤17 years	−5.38% (−6.43 to −4.32), *P*<.001	2.48% (1.66 to 3.30), *P*<.001
18-64 years	−1.07% (−2.74 to 0.62), *P*=.21	−1.34% (−2.63 to −0.07), *P*=.04

#### Sunshine Hours and the Risk of Varicella Incidence

With a 14-day lag, the exposure-response curve showing the relationship between sunshine hours and varicella risk is nonlinear. The risk of varicella increases with more sunshine hours, and this correlation is statistically significant (*P*=.007). Using a segmented linear regression function to assess the impact of sunshine hours on varicella incidence risk, a cutoff value of 1.83 hours was identified. Further analysis revealed that when sunshine duration is ≤1.83 hours, each 1-hour decrease results in a 6.05% decrease in varicella incidence risk (95% CI −12.02 to 0.33, *P*=.06), although this correlation is not statistically significant. Conversely, when sunshine duration exceeds 1.83 hours, each 1-hour increase leads to a 1.30% increase in varicella incidence risk (95% CI 0.62-2.00, *P*=.007). Analysis of gender and age subgroups indicates that sunshine duration has a greater impact on females than on males, and it has the most significant effect on adults aged 18-64 years (Table 7).

**Table 7:** Percentage change in the risk of varicella, its 95% CI, and *P* value.

Variable	Sunshine hours≤1.83 h	Sunshine hours>1.83 h
Total	−6.05% (−12.02 to 0.33), *P*=.06	1.30% (0.62 to 2.00), *P*=.007
Male	−2.96% (−11.56 to 6.50), *P*=.53	1.31% (0.48 to 2.15), *P*=.002
Female	−9.30% (−17.16 to −0.69), *P*=.04	1.30% (0.43 to 2.18), *P*=.003
≤17 years	−4.44% (−11.88 to 3.62), *P*=.27	0.99% (0.19 to 1.80), *P*=.02
18-64 years	−12.83% (−24.81 to 1.11), *P*=.07	2.41% (1.27 to 3.57), *P*<.001

#### Relative Humidity and the Risk of Varicella Incidence

With a 21-day lag, the exposure-response curve depicting the relationship between relative humidity and varicella risk shows a nonlinear pattern. As relative humidity increases, the risk of varicella initially decreases and then gradually increases, with this correlation being statistically significant (*P*<.001). A segmented linear regression function was used to assess the impact of relative humidity on varicella incidence risk, identifying a cutoff value of 57.182%. Further analysis revealed that when relative humidity is ≤57.182%, each 1% decrease leads to a 1.11% decrease in varicella incidence risk (95% CI −1.28 to 0.93, *P*<.001). Conversely, when relative humidity exceeds 57.182%, each 1% increase results in a 2.05% increase in varicella incidence risk (95% CI 1.26 to 2.84, *P*<.001). Subgroup analysis based on gender and age indicates that relative humidity presents a higher varicella risk for women compared with men. Additionally, when humidity exceeds 57.18%, the risk of varicella is greater in children under the age of 17 years (Table 8).

**Table 8:** Percentage change in the risk of varicella, its 95% CI, and *P* value.

Variable	Relative humidity≤57.18%	Relative humidity>57.18%
Total	−1.11% (−1.28 to −0.93), *P*<.001	2.05% (1.26 to 2.84), *P*<.001
Male	−1.28% (−1.49 to −1.07), *P*<.001	1.47% (0.48 to 2.46), *P*=.003
Female	−0.90% (−1.13 to −0.68), *P*<.001	2.75% (1.76 to 3.74), *P*<.001
≤17 years	−1.28% (−1.49 to −1.08), *P*<.001	2.72% (1.79 to 3.66), *P*<.001
18-64 years	−0.53% (−0.85 to −0.22), *P*=.001	−0.51% (−2.09 to 1.08), *P*=.53

#### Surface Mean Temperature and the Risk of Varicella Incidence

With a 21-day lag, the exposure-response curve illustrating the relationship between average ground temperature and varicella risk shows a nonlinear pattern. As average ground temperature increases, the risk of varicella initially rises and then declines, with this correlation being statistically significant (*P*<.001). A segmented linear regression function was used to assess the impact of average ground temperature on varicella incidence risk, identifying a cutoff value of 22.4°C. Further analysis reveals that when the average ground temperature is ≤22.4°C, each 1°C decrease leads to a 1.36% increase in varicella incidence risk (95% CI 0.96-1.75, *P*<.001). Conversely, when the average ground temperature exceeds 22.4°C, each 1°C increase results in a 2.14% decrease in varicella incidence risk (95% CI −2.61 to 1.66, *P*<.001). Subgroup analysis based on gender and age indicates that the impact of temperature changes on varicella incidence does not significantly differ between genders (*P*<.001). However, there is a greater impact on children under 17 years compared with individuals aged 18-64 years. The effect on the latter group was not statistically significant (*P*=.76; Table 9).

**Table 9:** Percentage change in the risk of varicella and its 95% CI and *P* value.

Variable	Surface mean temperature≤22.4°C	Surface mean temperature>22.4°C
Total	1.36% (0.96 to 1.75), *P*<.001	−2.14% (−2.61 to −1.66), *P*<.001
Male	1.46% (0.97 to 1.94), *P*<.001	−1.99% (−2.61 to −1.37), *P*<.001
Female	1.23% (0.72 to 1.75), *P*<.001	−2.31% (−2.97 to −1.65), *P*<.001
≤17 years old	1.72% (1.26 to 2.18), *P*<.001	−3.43% (−4.00 to −2.86), *P*<.001
18-64 years old	0.11% (−0.61 to 0.83), *P*=.76	1.42% (0.50 to 2.34), *P*=.002

#### Evaporation and the Risk of Varicella Incidence

With a 16-day lag, the exposure-response curve illustrating the relationship between evaporation and varicella risk is nearly linear. As evaporation increases, the risk of varicella gradually decreases, with this correlation being statistically significant (*P*<.001). Consequently, a linear term was used in the model to assess the impact of average evaporation on varicella risk. The results indicate that each 1 mm increase in evaporation corresponds to a 3.95% decrease in varicella risk (95% CI −5.04 to 2.85, *P*<.001). Notably, there is a significant impact on varicella risk in children under the age of 17 years, with a decrease of 4.83% (95% CI −6.09 to 3.55, *P*<.001; Table 10).

**Table 10:** Percentage change in the risk of varicella, its 95% CI, and *P* value.

Variable	Evaporation (mm)
Total	−3.95% (−5.04 to −2.85), *P*<.001
Male	−3.75% (−5.07 to −2.41), *P*<.001
Female	−4.20% (−5.75 to −2.80), *P*<.001
≤17 years	−4.83% (−6.09 to −3.55), *P*<.001
18-64 years	−1.01% (−2.89 to 0.91), *P*=.3

#### Wind Velocity and the Risk of Varicella Incidence

With a 16-day lag, the exposure-response curve depicting the relationship between wind speed and varicella risk is nearly linear. As wind speed increases, the risk of varicella gradually decreases, with this correlation being statistically significant (*P*<.001). Consequently, a linear term was used in the model to evaluate the impact of average wind speed on varicella incidence risk. The results indicate that for every 1 m/s increase in daily wind speed, the risk of varicella incidence decreases by 3.95% (95% CI −7.61 to −0.16, *P*=.04). Notably, there is a significant impact on varicella risk for adults aged 18-64 years, with a reduction of 10.48% (95% CI 3.59 to 17.82, *P*=.002; Table 11).

**Table 11:** Percentage change in the risk of varicella and its 95% CI and *P* value.

Variable	Wind velocity (s/m)
Total	−3.95% (−7.61 to −0.16), *P*=.04
Male	−4.98% (−9.35 to −0.41), *P*=.03
Female	−2.76% (−7.45 to 2.16), *P*=.27
≤17 years	−8.20% (−12.28 to −3.94), *P*<.001
18-64 years	−10.48% (3.59 to 17.82), *P*=.002

## Discussion

### Principal Findings

From 2011 to 2018, Wuxi city had the highest incidence of varicella in Jiangsu Province [21]. Since December 2018, the city has included the varicella vaccine in its routine immunization program, providing 2 free doses to eligible resident children [22]. Despite a reduction in varicella incidence in 2019, with a reported rate of 174.04 per 100,000 population, Wuxi city’s rate remained significantly higher than the national average of 70.14 per 100,000 population.

Previous studies have demonstrated that climatic factors such as temperature, humidity, rainfall, and wind speed significantly influence the occurrence of certain infectious diseases [23]. Our study has identified a nonlinear relationship between the risk of varicella (chickenpox) incidence and various climatic factors, including temperature, air pressure, precipitation, and relative humidity. Specifically, as temperature, surface mean temperature, and air pressure increase, the risk of varicella incidence initially rises and then declines. By contrast, with increasing precipitation and relative humidity, the risk of varicella initially decreases and then increases. Additionally, evaporation and wind speed are negatively correlated with varicella risk, while sunshine duration shows a positive correlation. In Wuxi city, varicella cases predominantly involve males, children, and students, aligning with trends observed in other regions of China [9]. However, in tropical regions, adolescents and young adults are more susceptible to varicella virus infection [11]. Additionally, our study identified a bimodal seasonal pattern of varicella incidence, consistent with patterns observed in Jinan [15], Shandong, and Lu’an [24], Anhui, China. This contrasts with studies conducted in Japan [17] and the United Kingdom [25], where the bimodal periodicity of varicella incidence in northern Japan transitions to a unimodal pattern in southern Japan, especially at lower latitudes. In the United Kingdom, varicella incidence also follows a bimodal pattern, with peaks observed in mid-March and June. These variations underscore the potential influence of location-specific climatic factors on the transmission of the VZV. Such discrepancies suggest that regional climatic conditions play a significant role in shaping the patterns of varicella incidence across different geographical areas.

Our research confirms previous findings regarding the relationship between temperature and varicella incidence risk. We observed that as both temperature and surface mean temperature increase, the risk of varicella incidence initially rises and then declines. This pattern is consistent with the results of a study conducted in Guangzhou, China, which also identified a ∩-shaped curve relationship between average temperature and varicella risk [8]. Our study found that when the average temperature and surface mean temperature are ≤20°C and 22.4°C, respectively, the risk of varicella incidence increases as these temperatures decrease, especially among male children under 17 years. The high incidence of varicella during the winter and spring seasons can be attributed to the significant temperature fluctuations during these periods. Temperature variations can weaken the immune system, increasing the risk of varicella infection. Specifically, the cold and dry weather in winter, combined with gradually rising temperatures and large temperature fluctuations in spring, creates favorable conditions for the transmission and reproduction of the VZV. During cold winter days, people tend to gather indoors more frequently, which increases interpersonal contact. As varicella is primarily transmitted through respiratory droplets and direct contact, such gatherings facilitate the spread of the virus. The study by Markus et al [26] further corroborates these findings, indicating that the VZV exhibits heightened replication efficiency at lower temperatures.

The exposure-response curve between air pressure and the risk of varicella exhibited a nonlinear relationship. Initially, as air pressure increased, the risk of varicella rose before eventually declining. Specifically, when the air pressure was ≤1011.28 hPa, a decrease in air pressure corresponded with an increased risk of varicella. These findings align with results from a study conducted in Guangzhou, China, which also demonstrated a similar pattern in the relationship between air pressure and varicella risk [8]. Although a clear causal relationship between changes in air pressure and the incidence of varicella has not been widely confirmed, variations in air pressure may indirectly influence the incidence of varicella by affecting human physiological functions and the immune system.

Our study found that the “exposure-response” curves for precipitation and relative humidity in relation to varicella incidence risk were consistent, both showing a nonlinear pattern. As precipitation and air humidity increased, the risk of varicella incidence initially decreased and then increased, particularly among children under 17 years. A study in Jinan also reported a positive correlation between varicella incidence and rainfall [15]. Another study showed that rainfall can indirectly influence varicella incidence by altering relative humidity and human activities [27]. As a nonlipid-coated virus, the VZV is more likely to survive and spread under specific humidity conditions. Relevant studies have shown that the risk of varicella peaks at 69.5% relative humidity [28]. This suggests that the varicella virus can maintain its activity and increase the risk of infection in high-humidity environments. Therefore, during rainy weather or when the varicella virus is particularly active, it is important to enhance cleanliness and air circulation in the environment. Methods such as opening windows for ventilation, using ultraviolet radiation, and other disinfection techniques can help reduce the risk of varicella virus transmission.

Our study found a positive correlation between sunshine duration and the risk of varicella, which aligns with findings from a study conducted in Jinan. Previous research has indicated that differences in varicella incidence between tropical and temperate regions are primarily influenced by sunshine hours and ultraviolet radiation [29]. A study conducted in Poland found that solar ultraviolet radiation during the summer might inhibit cellular immunity, leading to an increased incidence of herpes zoster [30]. Additionally, solar radiation may impact vitamin D metabolism and the host’s immune system, thereby elevating the relative risk of varicella [31]. Biological evidence indicates that vitamin D can inhibit human natural killer cells, which are crucial for innate defense against viral infections [32].

The associations between wind speed, evaporation, and varicella incidence were nearly linear. As wind speed and evaporation increased, the risk of varicella incidence gradually decreased. Wind affects the suspension time and diffusion distance of the virus. Sabariego et al [33] demonstrated that higher wind speeds can dilute local virus concentrations due to the resuspension phenomenon.

Additionally, global climate change, extreme weather events, population movements, changes in air pollutant concentrations, and vaccination rates may impact varicella incidence [34]. A systematic review of Latin America and the Caribbean found that the incidence of varicella has been increasing due to urbanization and higher population density [35].

### Limitations

This study has several limitations that should be noted. First, as an ecological study, it may be subject to ecological fallacies. The meteorological data used represent the overall averages for Wuxi city, and cannot be matched precisely to the specific meteorological conditions experienced by each case. As a result, accurate individual exposure levels could not be determined. This means that the research results may not be directly applicable to individual-level inferences. Second, due to the limited monitoring data available, the model only considered meteorological factors. Other potential influences on varicella incidence, such as host susceptibility, population density, air pollutants, and socioeconomic factors, were not included in the analysis. These limitations suggest that while the research offers valuable insights into the relationship between meteorological factors and varicella, further studies are needed to address these limitations and provide stronger data to support varicella prevention and control strategies. Additionally, this study is confined to environments similar to subtropical climates or urban areas and may not be generalizable to other regions.

### Conclusions

In this study, we identified significant correlations between varicella incidence and specific climatic factors in Wuxi from 2010 to 2019. Notably, varicella cases increased during months with high temperatures and humidity. By monitoring and analyzing meteorological data, we can predict trends and peak periods of varicella incidence, allowing for timely issuance of early warning information to alert relevant departments and the public to take preventive measures. This approach can help reduce varicella incidence and mitigate the impact of outbreaks on socioeconomic activities and daily life. While our study offers valuable insights, we recognize that the sample size and data collection period may limit the generalizability of our findings. Future research should explore broader geographical areas and longer timeframes to further validate our results and investigate other climatic factors that may affect varicella transmission.

## Abbreviations

CDC: Center for Disease Control and Prevention
GAM: Generalized Additive Model
NDSS: National Infectious Disease Surveillance System
VZV: varicella-zoster virus
WHO: World Health Organization

## Data Availability

The data sets generated or analyzed during this study are available from the corresponding author upon reasonable request.
